# An IMRT planning technique for treating whole breast or chest wall with regional lymph nodes on Halcyon and Ethos

**DOI:** 10.1002/acm2.14295

**Published:** 2024-02-09

**Authors:** Kareem Rayn, Ryan Clark, Klea Hoxha, Anthony Magliari, Jack Neylon, Michael H. Xiang, Dylan P. O'Connell

**Affiliations:** ^1^ Varian Medical Affairs Palo Alto California USA; ^2^ Department of Radiation Oncology University of California Los Angeles California USA

**Keywords:** breast IMRT, Ethos, Halcyon

## Abstract

**Purpose/Objective:**

Field size limitations on Halcyon and Ethos treatment machines largely preclude use of the conventional monoisocentric three‐field technique for breast/chest wall and regional lymph nodes. We present an alternative, IMRT‐based planning approach that facilitates treatment on Halcyon and Ethos while preserving plan quality.

**Materials/Methods:**

Eight breast and regional node cases (four left‐sided, four right‐sided) were planned for an Ethos machine using a 15–17 field IMRT technique. Institutional plan quality metrics for CTV and PTV coverage and OAR sparing were assessed. Five plans (four right‐sided, one left‐sided) were also planned using a hybrid 3D multisocenter technique. CTV coverage and OAR sparing were compared to the IMRT plans. Eclipse scripting tools were developed to aid in beam placement and plan evaluation through a set of dosimetric scorecards, and both are shared publicly.

**Results:**

On average, the IMRT plans achieved breast CTV and PTV coverage at 50 Gy of 97.9% and 95.7%, respectively. Supraclavicular CTV and PTV coverages at 45 Gy were 100% and 95.5%. Axillary lymph node CTV and PTV coverages at 45 Gy were 100% and 97.1%, and IMN CTV coverage at 45 Gy was 99.2%. Mean ipsilateral lung V20 Gy was 19.3%, and average mean heart dose was 1.6 Gy for right‐sided cases and 3.0 Gy for left‐sided. In comparison to the hybrid 3D plans, IMRT plans achieved higher breast and supraclavicular CTV coverage (99.9% vs. 98.6% and 99.9% vs. 93.4%), higher IMN coverage (99.6% vs. 78.2%), and lower ipsilateral lung V20 Gy (19.6% vs. 28.2%).

**Conclusion:**

Institutional plan quality benchmarks were achieved for all eight cases using the IMRT‐based planning approach. The IMRT‐based planning approach offered superior conformity and OAR sparing than a competing hybrid 3D approach.

## 1 INTRODUCTION

Outside of skin cancer, breast cancer is the most commonly diagnosed cancer in women, constituting 30% of all new female cancers each year. Incidence rates of breast cancer have been increasing by about 0.5% each year. The American Cancer Society estimates that 287,850 new cases of invasive breast cancer and 51,400 ductal carcinoma in situ will be diagnosed in women in 2022.^1^ Meta‐analysis of individual patient data by Early Breast Cancer Trialists' Collaborative Group (EBCTCG) found that following breast conserving surgery with radiotherapy halves the rate of breast cancer recurrence while also reducing the rate of breast cancer death by about one‐sixth. In addition, post‐mastectomy radiation therapy (PMRT) has been shown to decrease locoregional recurrence and improve overall survival, particularly in patients demonstrating high‐risk pathological features such as positive lymph nodes, positive surgical margins, and inflammatory breast cancer.^2^

Radiotherapy planning for breast cancer patients is technically challenging due to anatomical variations of the breast/chest wall, organ motion, and setup reproducibility. The use of tangential beams in a 3‐dimensional conformal radiation therapy (3DCRT) technique can avoid a low dose region in the ipsilateral lung but is associated with poor conformity, homogeneity, and hot spots outside the target volume. IMRT and VMAT can improve conformity but result in lower dose to the lung and contralateral breast, and require more monitor units and hence increased delivery time.^3^ The most commonly used treatment planning method for adjuvant radiation for whole breast or chest wall with regional nodes is a monoisocentric, 3D field‐in‐field technique using tangents and an anteroposterior supraclavicular field. A study conducted by Morris et al., outlines a 3D field‐in‐field planning technique in the Halcyon linear accelerator. In this study, the patients were treated via tangential beams with only the breast as target, while the cohort used in our study received radiotherapy to the regional lymph nodes as well.^4^

Halcyon (Varian Medical Systems, Palo Alto, CA, USA) treatment delivery platform includes a 6X flattening filter free (FFF) photon‐only linac, mounted on a ring structure with a 100 cm diameter bore. Current Halcyon systems can include kV CBCT, and two‐isocenter automatic feathering which allows the exceeding of the 28 × 28 cm^2^ aperture constraint. Due to faster gantry rotation speeds and MLC leaf speeds of 5.0 cm/s, the Halcyon treatment delivery platform delivers faster treatments than conventional C‐arm linacs. Halcyon systems also require daily image guidance due to the lack of light field and optical distance indicators.

Traditional C‐arm linacs allow for larger treatment fields with a single isocenter due to the increased maximum field size. While existing techniques for planning whole breast irradiation with a C‐arm using only tangential fields can be directly replicated on Halcyon platforms, treating the whole breast and regional nodal areas can be challenging due to the limited longitudinal field size of 14 cm from the isocenter when using a half‐beam block. This limitation also applies to the Ethos machine, which uses the same hardware platform as Halcyon. Kim et al.^5^ were the first to address this challenge in the literature, publishing a report describing a technique using multiple isocenters to cover the entire volume with a supraclavicular and four total tangent fields. The authors’ proposed technique closely replicates the dose distribution of a conventional 3D conformal treatment plan while leveraging the automated dose feathering and multi‐isocenter delivery capabilities of the Halcyon platform. Alternatively, the Halcyon/Ethos platform presents a compelling opportunity for investigating the feasibility and benefits of IMRT‐based planning for breast/chest wall and regional nodes.

In July 2022, ASTRO Choosing Wisely withdrew a 2013 recommendation against the use of routine IMRT for whole breast radiotherapy in patients with breast cancer.^6^ Proponents of IMRT in this setting argue that it allows for decreased radiation dose to the heart and allows for more homogenous dose distribution which may have a favorable impact on skin toxicity and cosmetic outcomes.^7^ Opponents of IMRT argue that it causes portions of the heart, lungs, and contralateral breast to receive additional low doses of radiation, which may increase risk of late effects including cardiac events, pulmonary disease, and secondary malignancy. However, there is lack of prospective data comparing late effects between alternative radiotherapy approaches.

In this study, we describe an IMRT‐based planning approach for Halcyon/Ethos with favorable dosimetry. Additionally, we developed an Eclipse plugin to automate multi‐field IMRT beam arrangements and created dosimetric scorecards using piecewise linear functions to measure individual quality metrics and assist in plan evaluation.

## 2 METHODS

### 2.1 Patient population

Eight patients previously treated at one institution were included in this retrospective study. Four patients had right‐sided disease (three invasive ductal carcinoma [IDC], one invasive lobular carcinoma [ILC]) and four patients left‐sided (3 IDC, 1 ILC). Two patients underwent lumpectomy while the remaining six underwent mastectomy and reconstruction prior to radiation therapy. Post‐mastectomy patients were treated with a custom‐printed 0.5 cm bolus (FlexiBol, .decimal, Sanford, Florida). Median age was 49 years. For all cases, 50 Gy in 25 fractions was prescribed to the breast or chest wall with 45 Gy to the regional nodes, including internal mammary lymph nodes.

### 2.2 Immobilization and simulation

All patients were positioned using a C‐Qual M adjustable breast board (Civco Radiotherapy, Coralville, Iowa) with arms overhead and the board angle set to 7.5°.

The contralateral elbow is the most likely collision point with Halcyon/Ethos breast radiation therapy. Two distance measurements (distance from midline to the contralateral elbow and vertical distance from the couch top to the contralateral elbow) taken at the time of simulation allows the planner to localize the elbow despite not being captured in the CT scan.^7^

Free‐breathing CT scans were acquired for all patients using a Biograph 64 (Siemens Healthineers; Erlangen, Germany). Deep inspiratory breath hold was not utilized. Images were reconstructed at 1.5 mm slice thickness.

### 2.3 Treatment planning

Clinical target volumes (CTVs) were contoured by the physician using only the CT simulation image in accordance with institutional guidelines. The breast PTV is then created by adding a 5 mm margin to the breast CTV. When the breast is being treated the PTV is pulled back 5 mm from the skin surface to match the CTV. If the chest wall is treated the PTV is not pulled back. Organs at risk (OARs) were subsequently contoured by the planner, including the contralateral breast, lungs, heart, esophagus, trachea, ipsilateral humeral head, skin, liver, and stomach.

#### 2.3.1 Multi‐isocenter 3D conformal

Five of the eight patients in this study (four with right‐sided cancers and one left‐sided) were planned using the multi‐isocenter hybrid 3D conformal technique described by Kim et al. in,^5^ which will subsequently be referred to as MI3D in this work. The MI3D technique aims to replicate the conventional monoisocentric half‐beam block 3D conformal breast and regional nodes planning approach as closely as possible on Halcyon/Ethos while using the multi‐isocenter delivery functionality with automated dosimetric feathering functionality as a workaround for the limited field size.

To summarize Kim et al.’s technique, a suitable isocenter position is chosen at the match line for the tangent and supraclavicular field border. A simple stand‐in plan is made for a C‐arm machine, such as a TrueBeam. In the stand‐in plan, the physician sets the tangent and supraclavicular apertures. Dose is calculated and the 50% isodose lines from the tangents and from the supraclavicular field are converted to structures for use as target volumes for Halcyon/Ethos planning.

A Halcyon/Ethos plan for the supraclavicular field is created using the same isocenter and field angles. The planner then adds a PAB field and uses irregular tissue compensation and fluence editing to achieve the desired dosimetry for the nodal targets.

Next, a second multi‐isocenter Halcyon plan is created with the initial isocenter at the match line and a second isocenter 8 cm inferior. The same tangent angles from the C‐arm stand‐in plan are used and the planner runs a simplified IMRT optimization to enable dosimetric feathering for the multi‐isocenter delivery.

The two plans are combined, and the planner then edits the field fluences, plan normalization, and field weights as necessary.

#### 2.3.2 IMRT

IMRT plans were generated for the five patients planned with the MI3D technique, as well as for three additional patients with left‐sided cancers.

Planning target volumes (PTVs) were generated by applying a 5 mm margin to the nodal and breast CTVs as described in Ref. 8. The exception was for the internal mammary node (IMN) CTV, for which 0 mm PTV margin was applied (i.e., CTV = PTV). PTVs were pulled back from the body surface by 5 mm for whole breast patients and by 2 mm for post‐mastectomy patients. The supraclavicular PTV was cropped off the esophagus expanded by a 3 mm PRV margin.

A 0.5 cm virtual bolus was created in Eclipse for post‐mastectomy patients. The virtual bolus was exported to .decimal and used to construct a patient‐specific physical bolus that was applied for treatment.

The IMRT plans used a single isocenter placed approximately at the centroid of the combined nodal and breast/chest wall PTVs. The isocenter positions were adjusted to ensure adequate clearance with the bore and remaining field size for adding flash to the tangential beams.

A standardized field arrangement was used for all patients. Beam angles were chosen using the following methodology:

- Set isocenter and medial tangent angle. The medial tangent angle should be carefully chosen to minimize dose to the heart and ipsilateral lung. All other fields are derived from this selection.
- Going medially, add additional field(s) in increments of the 8 degrees. The number of fields that can be added will depend on patient anatomy, avoiding entry through the contralateral breast.
- From the initial medial tangent field, add two more fields in increments of 8° going laterally.
- Add two more fields in increments of 16° going laterally.
- From the final medial field, add the first lateral field 96° laterally.
- Continuing laterally, add two more fields in increments of 16°.
- Continuing laterally, add five more fields in increments of 8°.
- For the PAB, add a field 24° from the final lateral field. This field angle can be modified by +/‐ 8° depending on the patient's anatomy.

Initially, a plan template with predetermined gantry angles was investigated, however due to variances in patient anatomy the fixed angle template did not always include ideal tangent beams. The above approach allows for a systematic selection of beam angles while accounting for anatomical variance.

The field arrangement was designed to simulate a volumetric arc therapy (VMAT) arc to allow for increased conformality and homogeneity. Additionally, the field placement procedure described above avoids generating directly opposed beams, which can degrade plan quality.^9^

VMAT was not used in this study because the patients were treated free‐breathing and respiratory motion would add substantial uncertainty. Such uncertainty also affects IMRT delivery,^10^ but the addition of flash to tangential fields can provide some mitigation.^11^ Flash was added by manually extending the fluence maps with the Eclipse skin flash tool. While adding flash to VMAT arcs is possible,^12^ the process is more straightforward with IMRT and therefore the IMRT approach was selected for this study.

**FIGURE 1:**
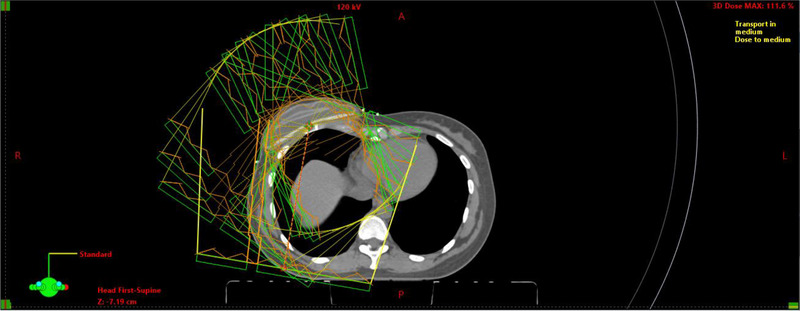
Example IMRT field arrangement for a right‐sided cancer case. The image shows the beam placement for a patient receiving treatment on the right side. The beam arrangement is intended to mimic a partial VMAT arc while instead using an IMRT technique. This allows for flash to be added to each field to mitigate deviations in the delivered dose due to respiratory motion. The number of fields was selected as a compromise between plan quality and delivery efficiency.

**FIGURE 2:**
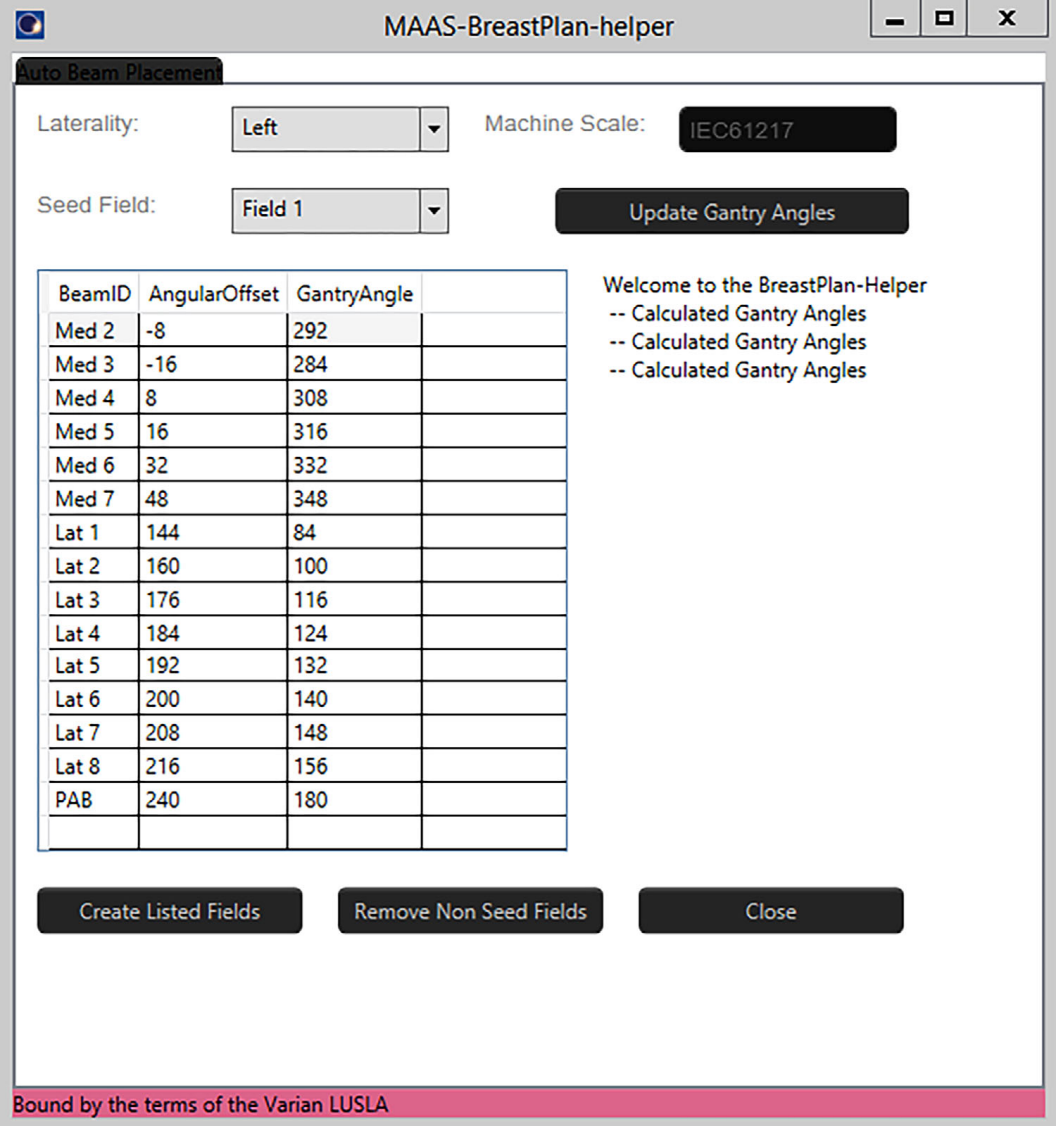
The BreastPlan helper interface. The figure shows an example of the Eclipse script‐automated beam placement after the user inputs a medical field angle. The script was developed to reduce the time spent on the beam placement process due to the large number of fields needed.

The large number of fields makes beam placement a time‐consuming process. To alleviate this, an Eclipse script was developed to automate beam placement after the initial medial tangent field is selected. Figure 1 shows the BreastPlan helper interface provided by the script and Figure 2 shows an example of the beam placement for a patient receiving treatment on the right side. The script is available on the Varian Medical Affairs website (https://github.com/Varian‐MedicalAffairsAppliedSolutions/MAAS‐BreastPlan‐helper). In addition to the script, there will be a case example hosted on the website as well (https://medicalaffairs.varian.com/lt‐chestwall‐ln‐50gy‐ucla‐imrt2).

IMRT plans were optimized using standardized objective templates for left‐sided and right‐sided targets. Objective templates are shared publicly on the Varian Medical Affairs website. Optimization was done using the Eclipse Photon Optimizer V15.6.1 and calculation was performed with the AcurosXB algorithm version 15.6.1. Heterogeneity in the resulting dose distributions was managed by running repeated intermediate dose optimizations, contouring hot spots, adding objectives to limit the maximum dose in those regions and in some cases by manually editing the beam fluence. After a satisfactory plan was generated, flash was manually added to all tangential fields with the Eclipse skin flash tool. Additional plan quality can often be found by continuing the previous optimization with intermediate dose after extending fluence into air for skin flash.

#### 2.3.3 Patient specific quality assurance

Quality assurance measurements for both the MI3D and IMRT plans were acquired before treatment for all patients. An Octavius 1500 array and octagonal phantom (PTW, Freiburg, Germany) were used. Plans were delivered using a fixed gantry angle of 0°. Measured planar dose distributions were compared to calculated distributions using the 3%/2 mm gamma criteria.^13^, ^14^

#### 2.3.4 Plan evaluation tool: Dosimetric scorecard

**FIGURE 3:**
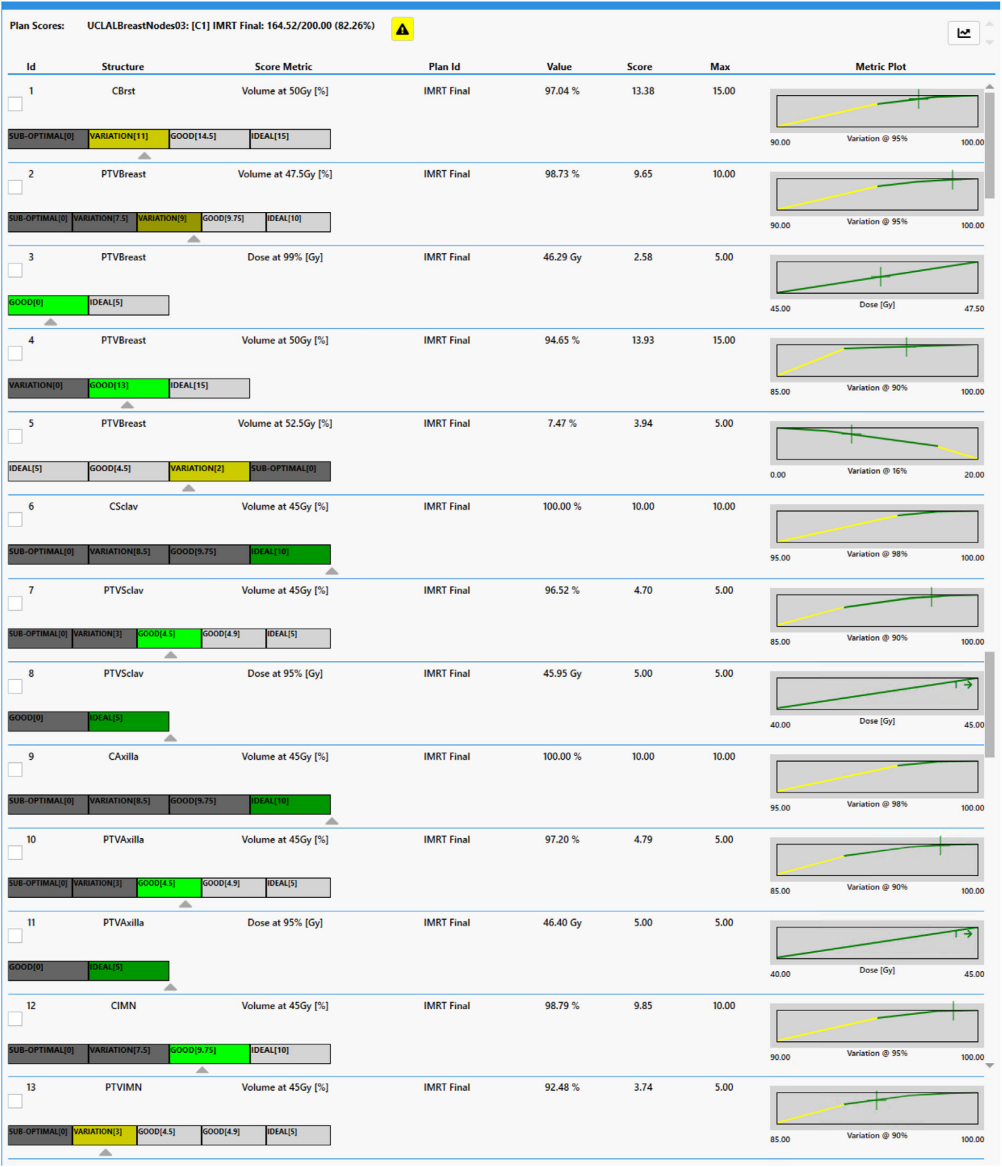
Example left‐sided breast case applying IMRT scorecard being applied to a left breast case. The scorecard shows a left‐sided breast node treatment case focusing on some of the IMRT plan quality metrics compared across the selected eight patient cases. The PlanScoreCard Eclipse Scripting Application Programming Interface tool (Varian Innovation Center GitHub) was used to generate the above scorecard.

Dosimetric scorecards were used to guide the planning process. The PlanScoreCard Eclipse Scripting Application Programming Interface tool, available free on the Varian Innovation Center GitHub, was used to create scoring metrics and score plans. These dosimetric scorecards use established scoring methodology of multiple piecewise linear score functions which measure specific plan quality metrics.^15^ The dosimetric scorecard, once established, allows for an objective measure of plan quality. An example of a dosimetric scorecard for a left‐sided case treatment is shown in Figure 3.

Dosimetric scorecards are scoring mechanisms that help to objectively quantify treatment plan quality. They operate by assigning points to competing metrics that represent the physician's preferences or institutional constraints. Each function spans a DVH value range of two or more values: the starting, failing (0 points), through the maximum, but often purposely unachievable, point value. Additional intermediate points can be added in between the failing point and the unachievable maximum to represent expected DVH values or previously reported ones. The dosimetric scorecards include visual diagrams of the score ranges from the failing point to the unachievable maximum, color‐coded to the preference of the user. The piecewise linear function graphs are generated for each metric using Volume [%] or Dose [cGy] for the x‐axis and the score achieved for that metric for the y‐axis (shown in detail in Figure 6). The graphs are also color‐coded to represent the score ranges/values in which a plan will fail (red), be acceptable (yellow), or reach the clinical benchmark (green), the maximum score, marked with blue, is the unachievable point value. Historical plan quality data collected for breast patients treated at our institution was used to inform the scorecard design.

#### 2.3.5 Plan evaluation: MI3D vs. IMRT

Two separate dosimetric scorecards, one for left and one for right‐sided targets, were generated to evaluate only the IMRT plans. Scorecards with relaxed quality metrics to account for different goals, such as planning to CTV coverage versus PTV coverage, were also created to assess the differences in dosimetry between the MI3D and IMRT for the five patients planned using both techniques. The specific dosimetric piecewise linear score functions used in this study were derived from institutional plan quality metrics. The scorecard tool and all scorecards used in this study are publicly shared online on the Varian Medical Affairs website. Statistical significance between quality metrics was assessed via p‐values calculated using the Rank Sum Test.

## 3 RESULTS

### 3.1 Patient specific quality assurance

All eight IMRT plans passed patient specific QA using an institutional minimum pass rate of 95% and the recommended criteria of 3%/2 mm with a 10% dose threshold.^13^ The mean gamma pass rate was 99.4% with a minimum pass rate of 98.0% and a maximum pass rate of 100.0%.

### 3.2 Dosimetric endpoints

**FIGURE 4:**
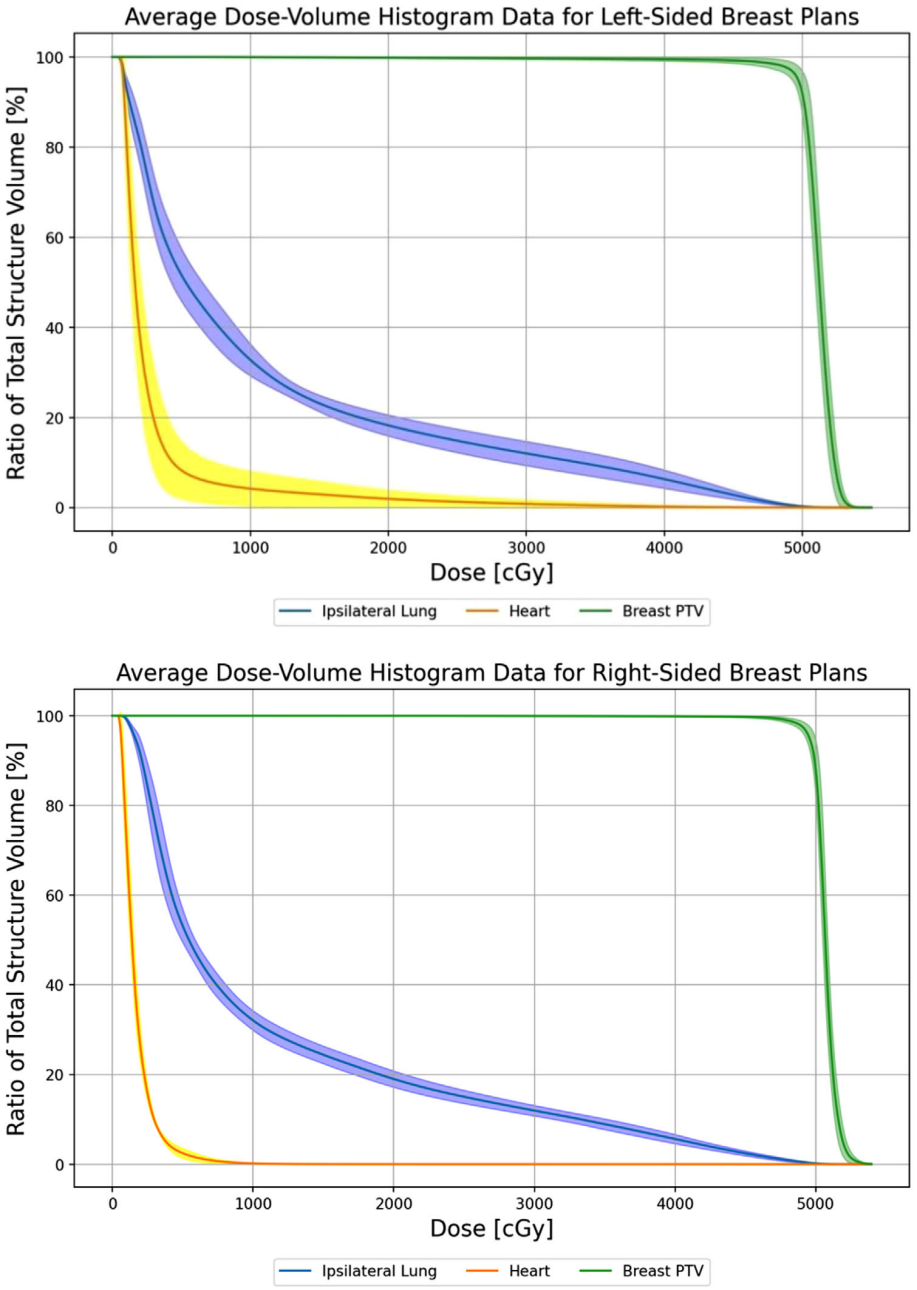
Average dose‐volume histogram data for left and right‐sided breast plans. The average DVH graphs for ipsilateral lung, heart, and breast PTV along with the standard deviation cloud around them are plotted for the left and right‐sided breast plans separately.

Plan scorecards derived from institutional plan quality benchmarks were used to quantitatively assess dosimetry. Dose constraints and average metric values for the eight IMRT plans are listed in Table 1. All plans were able to meet institutional plan quality guidelines. The IMRT technique excelled at achieving high IMN coverage while still sparing the heart, with an average mean heart dose of 3.0 Gy for left‐sided cases. Figure 4 shows a comparison of the average DVH graphs for left and right‐sided breast IMRT plans for breast PTV, mean heart dose, and ipsilateral Lung. The plots also include the standard deviation for each metric.

**TABLE 1:** IMRT plan quality metrics across eight patients.

Structure	Plan metric	IMRT average	Spread (min—max)	Clinical criteria
Breast CTV	V50 Gy (%) V52.5 (%)	97.9 5.0	96.5–99.0 0.1–16.7	V45Gy ≥ 95–90%
Breast PTV	V50 Gy (%) V52.5 (%)	95.7 5.0	93.8–97.0 0.2–15.8	V45Gy ≥ 95–90%
Sclav CTV	V45 Gy (%) V47.25 (%)	100 99.8	99.7–100 99.2–100	V45Gy ≥ 95–90%
Sclav PTV	V45 Gy (%) V47.25 (%)	95.5 85.6	94.5–97.0 85.6–94.1	V45Gy ≥ 95–90%
Axilla L1,L2,L3 CTV	V45 Gy(%) V47.25 (%)	100 99.6	99.9–100 98.9–100	V45Gy ≥ 95–90%
Axilla L1,L2,L3 PTV	V45 Gy (%) V47.25 (%)	97.1 92.5	95.6–99.4 87–98.2	V45Gy ≥ 95–90%
IMN CTV	V45 Gy (%) V47.25 (%)	99.2 96.5	96.8–100 83.1–99.8	V45Gy ≥ 90%
Heart	Mean (Gy)	Left: 3.0 Right: 1.6	Left: 1.6–4.3 Right: 1.5–1.9	Mean ≤ 4–5 Gy
	D.03 cc (Gy)	23.6	6.3–52.8	D.03 cc ≤ 53 Gy
Ipsilateral Lung	V20 Gy (%) V5 Gy (%)	19.3 51.0	15.8–23.1 44.4–61.7	V20 Gy ≤ 30–35% V5 Gy ≤ 50–55%
Contralateral Lung	V5 Gy (%)	0.2	0–0.6	V5Gy ≤ 10–15%
Esophagus	D.03cc (Gy)	18.6	12.4–25.5	D.03 cc ≤ 50 Gy
Contralateral Breast	D.03cc (Gy)	3.7	2.7–4.7	D.03cc ≤ 3.1–4.96 Gy

*Note*: The listed structures and dose constraints were used to evaluate the IMRT plans created for the eight selected cases. The average IMRT metrics across all the cases along with their spread (min—max) are reported as well. The utmost right of the table reports clinical constraints for all relevant metrics.

The average scores for the left and right‐sided IMRT plans were 183 and 168, respectively, out of a maximum of 200 points on the dedicated IMRT scoring rubric.

Using the IMRT and 3D plan comparison scorecard, the average score across the 5 five cases planned with both techniques was 119 for the MI3D plans and 138 for the IMRT plans out of a maximum of 153 points.

### 3.3 MI3D versus IMRT comparison

**FIGURE 5:**
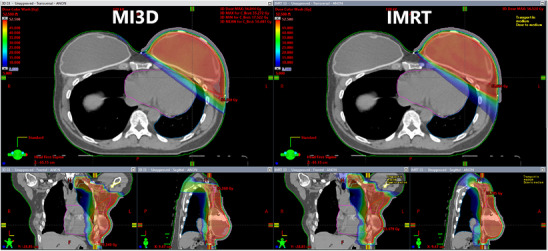
Dose distribution comparison between MI3D and IMRT techniques. The view shows an increase in high‐dose conformity for the IMRT plan accompanied with more low‐dose spillage.

**FIGURE 6:**
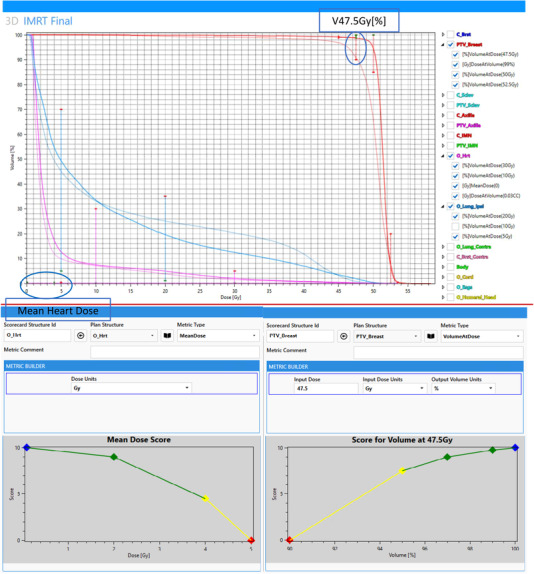
DVH view for both 3D and IMRT plans and score metric plot examples. The DVH view along with the scorecards were used to compare 3D and IMRT plans for an example left‐sided breast case. The structures included in the DVH view are the PTV, heart, and ipsilateral lung for both 3D (lighter) and IMRT (darker). The piecewise linear functions of the PTV and heart score metric plots were taken in the metric editor screen to show how the plots were generated. The colored marks on the DVH view show the range of values dictated by the scorecards that correspond to the piecewise linear graphs. The two example graphs are marked in the DVH view.

Overall, in comparison to the MI3D plans, the IMRT plans were able to achieve higher breast CTV coverage [*p*‐value = 0.00794], lower ipsilateral lung V20 Gy [*p*‐value = 0.00794] but there was a slight increase in the contralateral lung V5Gy [*p*‐value = 0.0476]. There was no statistical significance in the average mean heart dose [*p*‐value = 1.00] and ipsilateral lung V5Gy [*p*‐value = 0.222]. Figure 5 shows an example of the dose distribution comparison between an MI3D plan and an IMRT plan. An additional comparison is displayed in Figure 6 which shows a DVH view comparing MI3D and IMRT plans for an example left‐sided case as well as close‐up views of the piecewise linear functions used in scorecards. A summary of relevant dose constraints and average values for both techniques is provided in Table 2. The initial five patients were treated with the MI3D technique and replanned using the IMRT technique for comparison purposes. The IMRT technique compared favorably across this small cohort, offered improvements in planning efficiency, and reduced inter‐planner variability. Our institution adopted the IMRT planning technique for subsequent patients treated on Ethos.

**TABLE 2:** Relevant plan quality metrics comparison for MI3D versus IMRT.

Structure	Plan metric	MI3D average	IMRT average	MI3D median	IMRT median	*p*‐value
Breast CTV	V47.5 Gy (%)	98.6	99.99	99.2	100	0.00794
	V50 Gy (%)	68.8	98.3	69.3	98.5	0.00794
Sclav CTV	V45 Gy (%)	93.4	99.9	94.8	100	0.00476
Axilla L1,L2,L3 CTV	V45 Gy(%)	98.4	100	99.2	100	0.0317
IMN CTV	V45 Gy (%)	78.2	99.6	98.1	99.8	0.151
Heart	Mean (Gy)	1.93	2.09	1.53	1.6	1
	D.03 cc (Gy)	26.5	19.9	22.8	14.23	1
Ipsilateral Lung	V20 Gy (%)	28.2	19.6	28.9	19.6	0.00794
	V5 Gy (%)	47.0	50.0	46.1	49.6	0.222
Contralateral Lung	V5 Gy (%)	0.0	0.1	0	0.1	0.0476
Esophagus	D.03cc (Gy)	20.1	17.0	21.2	17.4	0.0952
Contralateral Breast	V5 Gy (%)	1.35	1.72	1.5	1.6	0.421

*Note*: A comparative summary of relevant dose constraints, average and median values for both techniques, MI3D and IMRT, as well as the *p*‐values obtained using the Rank Sum Test for four right‐sided cases and one left‐sided case.

### 3.4 Conformal versus IMRT plans

Our institution has been using in‐house software to track plan quality metrics for all of our breast patients treated in the past 3 years. We compared the metrics for the IMRT plans discussed in this study to average values across our large institutional dataset. All of the patients comprising the large dataset were treated with a traditional three‐field technique on C‐arm linacs. Table 3 provides a comparison. Both the IMRT plans and the 3DC plans comparably meet institutional constraints.

**TABLE 3:** Comparison between IMRT Plan Quality and historical institutional 3DC Population data.

Structure	Plan metric	IMRT average	Clinical criteria	3DC institutional population
Breast CTV	V50 Gy (%)	97.9 ± 1.07 %	V45Gy ≥ 95–90%	96.60 ± 2.89%
Sclav CTV	V45 Gy (%)	100 ± 0.12 %	V45Gy ≥ 95–90%	98.95 ± 1.69%
IMN CTV	V45 Gy (%)	99.2 ± 1.04 %	V45Gy ≥ 90%	97.21 ± 9.49%
Heart	Mean (Gy)	2.30 ± 1.17 %	Mean ≤ 4–5 Gy	1.45 ± 0.80 Gy
Ipsilateral Lung	V20 Gy (%)	19.3 ± 2.32 %	V20 Gy ≤ 30–35%	26.46 ± 4.44%
Contralateral Lung	V5 Gy (%)	0.2 ± 0.21 %	V5Gy ≤ 10–15%	0.23 ± 1.06%
Contralateral Breast	D.03cc (Gy)	3.7 ± 0.78 Gy	D.03cc ≤ 3.1–4.96 Gy	11.51 ± 14.10 Gy

*Note*: Table 3 shows a comparison of plan quality metrics from the IMRT plans used for this study and the average values for traditional 3D conformal (3DC) plans for C‐arm machines collected by our institution over the last 3 years. Notably, the 3DC contralateral breast D.03cc is substantially higher than IMRT because achieving necessary coverage of the IMNs occasionally requires the field to abut the contralateral breast.

### 3.5 Number of IMRT fields

**FIGURE 7:**
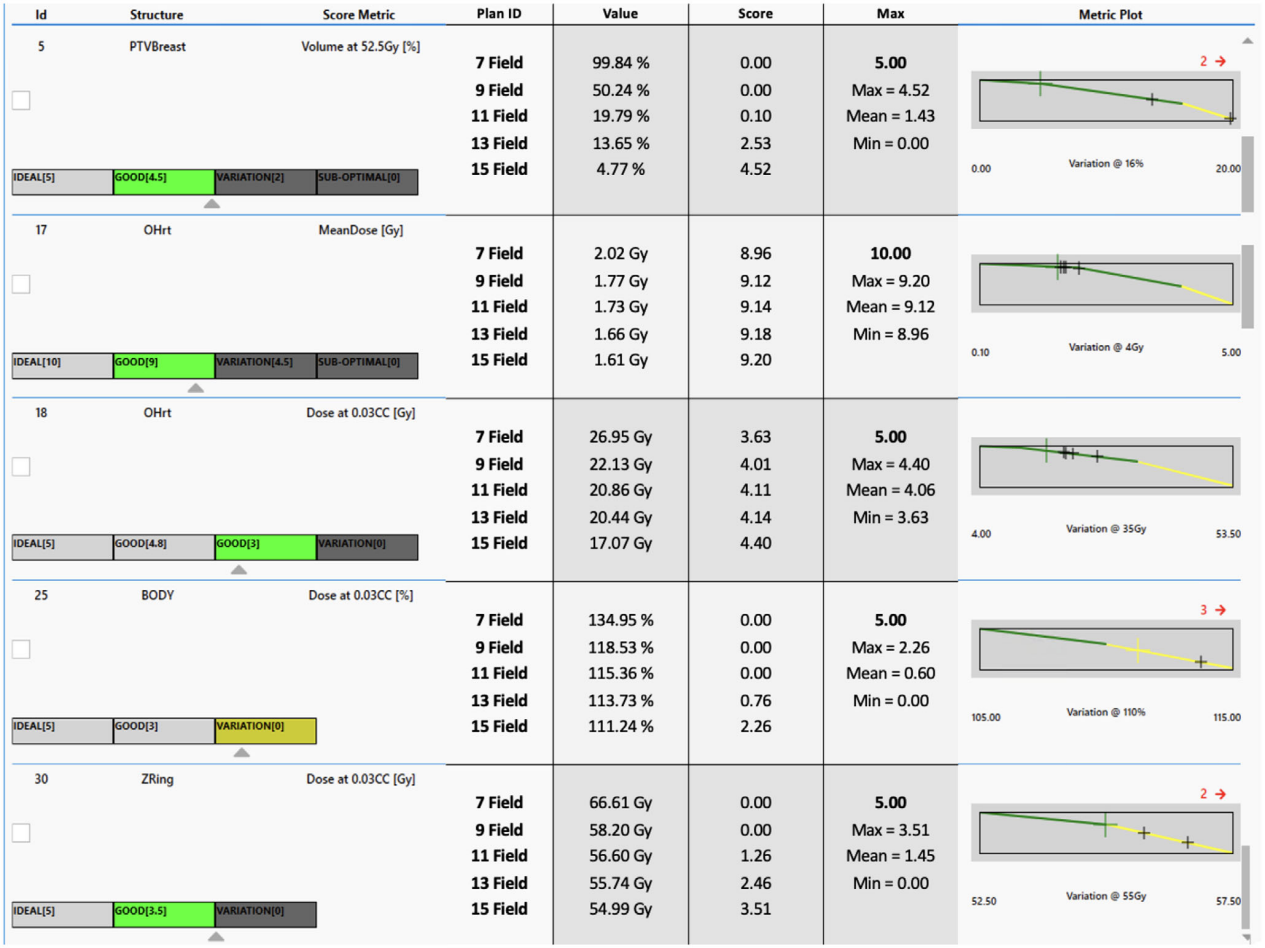
IMRT dosimetric scorecard demonstrating plan quality dependence on field number. The scorecard shows the relationship between plan quality and field number on an example left‐sided breast case. Listed are the dose metrics that most impacted plan score when evaluating the number of IMRT fields (7, 9, 11, 13, & 15) used. The dose metrics reported are PTV V105%, Heart D_mean_ and D_max_, Body D_max_ and PTV Ring D_max_.

The 15−17 field arrangement chosen for the IMRT plans was determined as an acceptable balance between plan quality, plan complexity, and delivery time. Figure 7 shows a scorecard of the dose metrics that most impacted plan score. One of the structures is the PTV Ring (Zring), defined as the total body structure excluding the total PTV volume and a 5 mm margin. The D.03cc of this structure was used as a measure of plan conformality. The effect of removing fields from the standard beam arrangement on plan quality was investigated in order to determine if delivery efficiency could feasibly be increased without degrading performance. For a single left‐sided case, multiple IMRT plans were generated using the same optimization objectives but with various numbers of fields. The left‐sided IMRT plan scorecard was used to assess how plan quality varied with the number of fields. Total scores out of 200 points were: 7 fields: 170, 9 fields: 168, 11 fields: 171, 13 fields: 174, and 15 fields: 180. Removal of fields from the proposed arrangement resulted in a decline in plan quality.

## 4 DISCUSSION

In this study, we described an IMRT‐based planning technique for treating whole breast or chest wall and regional nodes on the Ethos and Halcyon. Field size limitations on these platforms make the conventional monoisocentric, three‐field planning technique unworkable for most patients. The IMRT‐based approach described here produced plans achieving institutional plan quality benchmarks for coverage, ipsilateral lung V20 Gy, and mean heart dose. Additionally, the IMRT plans were compared to a previously published hybrid 3D multi‐isocenter approach and were able to achieve higher breast and supraclavicular coverage, higher IMN coverage. No statistically significant difference was noted in the mean heart dose between the two approaches. Notably, only five cases were used in the comparison between MI3D and the IMRT approach which is limiting.

One potential limitation of the comparison of our approach against MI3D is inter‐planner variability—the IMRT and MI3D plans were generated by different planners. The MI3D approach involves a substantial amount of manual fluence editing and the final plan is heavily dependent on the planner's skill level. Importantly, we believe that the IMRT approach here is more straightforward and can result in consistent high‐quality plans with less inter‐planner variance, particularly when the tools for beam placement and optimization objective templates provided are employed. An experienced planner can generate a complete plan in 3–4 h.

In the United States, 3DCRT remains the most prevalent treatment modality for breast radiotherapy.^16^ The usage of breast IMRT declined markedly following a 2013 Choosing Wisely publication^6^ by the American Society for Radiation Oncology (ASTRO) recommending against it.^17^ The Choosing Wisely campaign promoted evidence‐based, value‐driven care, suggesting that dosimetric improvement afforded by IMRT was not substantial enough to justify the increased cost and complexity, or that enough evidence of clinical benefits had not been detailed in the literature at the time to warrant its use.

Despite this, multiple studies have described the benefits of IMRT. In a large, prospective, multi‐institution study Jagsi et al.^18^ found a significant reduction in acute toxicity from IMRT over 3DCRT. In a review of literature on randomized controlled trials about IMRT in conservatively resected breast cancer, five randomized trials were identified by Buwenge et al. Patients treated with IMRT showed reduced acute toxicity according to all these studies. Additionally, two of these studies found that patients treated with IMRT also experienced less chronic radiation toxicity effects. Despite the comparable overall survival, disease‐specific survival, and freedom from ipsilateral breast tumor recurrence, studies support IMRT as one of the most heart‐sparing radiation therapy techniques for left‐sided breast cancer cases.^19^, ^20^

Two important issues related to IMRT for breast cancer are summarized by Kachnic et al.: its ability to provide better coverage of at‐risk nodal groups and to create improved dose gradients between the target volume and adjacent lung and heart, which could lead to improved outcomes or reduced toxicity. IMRT may be most beneficial for node‐positive breast cancer, especially when internal mammary nodal regions require treatment, since coverage of these nodes using non‐IMRT techniques is often inadequate, suggesting that IMRT may improve locoregional control in these situations.^9^, ^23^ Numerous phase II and retrospective studies on IMRT in breast cancer have demonstrated a decrease in acute radiation‐induced toxicity and reduced chronic breast edema, with comparable results in terms of local control and secondary malignancies when compared to standard techniques.^2^, ^21^, ^22^, ^23^ Critics of IMRT claim that it can lead to higher percentages of the heart and lungs being exposed to low doses of radiation, which could increase the risk of long‐term side effects such as cardiac events, pulmonary disease, and secondary malignancies. Nevertheless, there is insufficient data available to compare these late effects with those of alternative radiotherapy methods. This study demonstrates comparable low dose spread for IMRT and 3D plans with similar lung V5 dosimetric results (ipsilateral Lung V5 47% for 3D and 50% for IMRT [*p*‐value = 0.222], contralateral lung V5 0.0% for 3D versus 0.1% for IMRT [*p*‐value = 0.0476], and contralateral breast D5% 1.4 Gy for 3D and 1.7 Gy for IMRT [*p*‐value = 0.0952]). Another potential drawback of IMRT plans is the higher average delivery time compared to 3D plans; IMRT plans generated for our study required approximately 6 min to deliver all fields versus 2 min for both, a conventional monoisocentric 3D plan and a Halcyon MI3D approach. IMRT delivery time can be reduced by moving to a VMAT technique. A study conducted by Sun et al. reports an average delivery time of 2.18 ± 0.15 min for two whole‐arc Halcyon VMAT plans.^24^

In July 2022, citing recently published evidence related to IMRT for breast cancer, ASTRO rescinded the 2013 recommendation against its usage.^6^ Large scale, multicenter analyses in the literature, such as the work by Jagsi et al. and the rescinding of ASTRO's recommendation against breast IMRT demonstrate the viability of our proposed technique as a method to overcome machine limitations as well as a means of achieving superior plan quality.

Using the multifield technique described here, treatment plan quality with static gantry IMRT, with enough treatment fields, approaches the quality of VMAT while allowing for more robust treatment delivery due to the control of skin flash. Future planning software that can create VMAT apertures into the air to create skin flash during optimization would render this technique with multiple static fields unnecessary. Alternatively, use of commercial surface‐imaging systems with automated beam gating capabilities would allow for deep inspiration breath hold treatments, reducing or eliminating the need for skin flash.

## 5 CONCLUSION

Institutional plan quality benchmarks were achieved for all eight cases using the IMRT‐based planning approach, demonstrating the feasibility of treating whole breast or chest wall and regional nodes on an Ethos/Halcyon machine. The IMRT‐based planning approach described here offered superior conformity and OAR sparing than a competing hybrid 3D approach, with the tradeoff of increased low dose spill and delivery time. We have also developed and shared tools to available to make this technique easier to implement in the clinic.

## AUTHOR CONTRIBUTIONS

Kareem Rayn, MD: Co‐authored sections, directed all content, proofing all content. Ryan Clark, MS, CMD: Co‐authored sections, created figures, created most of the non‐clinical treatment plans, tested different beam arrangements and number of beams, co‐developed software tools used (materials), proofing all content. Klea Hoxha, BS: Contributed to running the gamma test and analysis, contributed to writing the manuscript. Anthony Magliari, MS, CMD: Co‐authored sections, directed all content, co‐developed software tools (materials), proofing all content. Jack Neylon, PhD: Contributed to the technical development of the Eclipse scripting tools. Michael H. Xiang, MD, PhD: Provided clinical data, supervised patient treatments, proofread and edited the manuscript. Dylan P. O'Connell, PhD: Contributed to the development of the study design, writing part of the manuscript.

## CONFLICT OF INTEREST STATEMENT

We disclose that any potential conflicts of interest could come from three of the authors (Kareem Rayn, Ryan Clark, Anthony Magliari) being employed by Varian Medical Affairs. Additionally, Michael H. Xiang and Dylan P. O'Connell have consulted for Varian, but their work was unrelated to the current manuscript. The study was not funded by the vendor.
